# High occurrence of antihistamine resistance in patients with recurrent idiopathic angioedema

**DOI:** 10.1186/s13601-019-0274-7

**Published:** 2019-07-12

**Authors:** Zonne L. M. Hofman, Nikki van West, C. Erik Hack, André C. Knulst, Coen Maas, Heike Röckmann

**Affiliations:** 1 Laboratory of Clinical Chemistry and Haematology, University Medical Center Utrecht, Utrecht University, Utrecht, The Netherlands; 2 Laboratory for Translational Immunology, University Medical Center Utrecht, Utrecht University, Utrecht, The Netherlands; 3 Department of Dermatology/Allergology, University Medical Center Utrecht, Utrecht University, Heidelberglaan 100, 3584 CX Utrecht, The Netherlands

**Keywords:** Angioedema, Idiopathic angioedema, Antihistamines, Omalizumab

## Abstract

Antihistamines are the most prescribed therapy in recurrent idiopathic angioedema, yet little is known about their efficacy. Herein, we report on clinical improvement with antihistamine therapy in 120 patients evaluating angioedema attack frequency. A high incidence (36%) of antihistamine refractory cases was observed. Forty percent of patients on antihistamine prophylaxis suffered from 1 or more angioedema attacks per month. Our findings stress the need for additional treatment options for recurrent idiopathic angioedema.

## Introduction

Angioedema is characterized by swelling of subcutaneous or mucosal tissue that may last up to 72 h and often recurs. Swellings can be disfiguring and lead to impaired functioning and quality of life [1]. Upper airway swellings require immediate medical care [2, 3]. Angioedema may be driven by bradykinin and/or mast-cell mediators including histamine. A disease classification is made based upon the underlying disease mechanism [4]. Bradykinin induced angioedema includes all types of hereditary angioedema, acquired C1-esterase inhibitor deficiency and angiotensin converting enzyme (ACE) inhibitor induced angioedema. Mast-cell mediator induced angioedema includes IgE mediated allergic angioedema and non-IgE mediated angioedema with urticaria. When no underlying cause can be identified, idiopathic angioedema is diagnosed [4]. The disease mediator of idiopathic angioedema is unknown but the disease is considered as part of the spectrum of chronic spontaneous urticaria and suspected to be mast-cell mediator induced [4, 5].

Antihistamines are first line therapy for idiopathic angioedema although their efficacy was never established in randomized control trials. Only two observational studies in a cohort of Italian angioedema patients report that 84% of patients are diagnosed as idiopathic histaminergic angioedema, i.e. respond to antihistamines [6, 7]. To what extent patients respond in terms of reduction in angioedema attack frequency and severity is not reported, leaving clinicians with limited information on the most commonly prescribed therapy for angioedema.

We undertook a retrospective evaluation of therapeutic management of recurrent idiopathic angioedema with antihistamines in a tertiary treatment center. Attack frequency and need for acute treatment during follow-up are reported, as well as usage of add-on therapy.

## Methods

We performed a retrospective analysis of medical records in all patients diagnosed with recurrent angioedema who visited our Dermatology/Allergology out-patient clinic between January 2008 and April 2017.

Patients were included when they experienced: recurrent spontaneous angioedema, duration of disease of > 6 weeks and had at least one follow-up visit to evaluate therapeutic management. Main exclusion criteria were other disease subtypes than idiopathic angioedema (e.g. angioedema with urticaria, hereditary angioedema, allergic angioedema, ACE-inhibitor induced angioedema and acquired C1-esterase inhibitor deficiency angioedema). Based on the treating physicians evaluation, antihistamine prophylaxis was started or increased in dose. After approximately 4 weeks, symptoms were evaluated and where necessary adjusted and followed-up. Various types of mainly second generation H1-receptor antagonists were prescribed. Clinical characteristics, including historical attack frequency, were collected at first visit and at evaluation of maximum prescribed therapy. Attack frequency was categorized as ≥ 1 per week, ≥ 1 per month but < 1 per week, ≥ 1 per year but < 1 per month and no further attacks. Improvement was defined as shift into a lower attack frequency category. Patients reporting no improvement or receiving add-on therapy such as omalizumab or cyclosporine, were defined as antihistamine-refractory or ‘non-responder’. To assess attack severity, necessity for acute treatment was scored based on treatment intensity ranging from: (additional) antihistamines and/or corticosteroids, adrenaline auto-injector, emergency medical care to hospital or intensive care admission. Graphpad Prism7.04 was used for statistical analysis. Groups were compared with Mann–Whitney test or Wilcoxon test in case of paired samples.

## Results

511 medical records were screened and 120 patients included. Patients’ characteristics are described in Table 1. Most common site of angioedema was the face (n = 105; 88%). Alarmingly, 28 patients (23%) reported to have experienced suspected laryngeal angioedema described as having dyspnea, difficulty swallowing or sudden changing voice.

**Table 1:** Clinical characteristics

	Total group (n = 120)
Female (%)	78 (65%)
Male (%)	42 (35%)
Mean age at first consult (range)	
All patients	51 (13–86)
Duration of disease (%)	
< 1 year	44 (37%)
1 to < 5 years	50 (42%)
5 to < 10 years	14 (12%)
10 years or >	12 (10%)
Family history with AE; first grade family member^a^ (%)	
Unknown	51 (43%)
No	59 (50%)
Yes	10 (8%)
Referral (%)	
General practitioner	63 (53%)
Secondary or tertiary treatment center	57 (48%)
Locations of AE attacks (%)	
Facial	105 (88%)
Oropharyngeal	93 (78%)
Laryngeal	28 (23%)
Abdominal	8 (7%)
Peripheral	37 (31%)

^a^Hereditary angioedema based on C1 esterase deficiency was excluded in patients with a positive family history by means of C4 screening

At first visit or during follow-up, 99 patients started on antihistamine prophylaxis or had a dose increase. 21 patients did not receive prophylaxis or their dose prescribed prior to first visit was not increased. The majority of patients (54%) was followed-up for > 6 months. Details on therapy and attack frequency in all patients are listed in Table 2.

**Table 2:** Prophylactic treatment, angioedema attack frequency and necessity for acute attack treatment at baseline and follow-up

	Baseline (n = 120)	Follow-up (n = 120)
Prophylactic treatment (%)		
Unknown	3 (3%)	0 (0%)
None	62 (52%)	18 (15%)
Antihistamine mono-therapy	45 (38%)	87 (73%)
Onefold daily dose	27 (23%)	17 (14%)
Twofold daily dose	11 (9%)	30 (25%)
Threefold daily dose	2 (2%)	7 (6%)
Fourfold daily dose	5 (4%)	21 (18%)
> Fourfold daily dose	0 (0%)	12 (10%)
Antihistamines + add-on	7 (6%)^a^	15 (13%)^c^
Other treatment	3 (3%)^b^	0 (0%)

	Baseline (n = 120)	All n = 120	No intervention n = 21^d^	Antihistamines only n = 99	+ Add-on n = 99
Attack frequency (%)					
Unknown	17 (14%)	15 (13%)	3 (14%)	11 (11%)	12 (12%)
≥ 1 per week	42 (35%)	15 (13%)	2 (10%)	17 (17%)	13 (13%)
≥ 1 per month	44 (37%)	24 (20%)	4 (19%)	23 (23%)	20 (20%)
≥ 1 per year	17 (14%)	30 (25%)	6 (29%)	23 (23%)	24 (24%)
No attack	0 (0%)	36 (30%)	6 (29%)	25 (25%)	30 (30%)

	Baseline (n = 120)	All = 120	No intervention n = 21	Antihistamines + add-on n = 99
Acute attack treatment (%)^e^				
Unknown	15 (13%)	18 (15%)	3 (14%)	15 (15%)
None	11 (9%)	51 (43%)	6 (29%)	45 (45%)
1. Antihistamines	25 (21%)	26 (22%)	8 (38%)	18 (18%)
2. Antihistamines and/or corticosteroids	14 (12%)	17 (14%)	2 (10%)	16 (16%)
3. Epipen	5 (4%)	3 (3%)	1 (5%)	2 (2%)
4. Urgent care center	43 (36%)	5 (4%)	2 (10%)	3 (3%)
5. Hospitalization or Intensive care	7 (6%)	0 (0%)	0 (0%)	0 (0%)

^a^Antihistamines combined with leukotriene antagonist (n = 2), corticosteroids (n = 3), H2 antagonist (n = 1), tranexamic acid (n = 1)

^b^Monotherapy with tranexamic acid (n = 2) or H2 antagonist (n = 1)

^c^Antihistamines combined with omalizumab (n = 8), omalizumab and tranexamic acid (n = 1), leukotriene antagonist (n = 1), cyclosporine (n = 1), tranexamic acid (n = 2), sulfalazine (n = 1), H2 antagonist (n = 1)

^d^No prophylaxis (n = 18) no antihistamine dose increase (n = 3)

^e^Overview of the most invasive reported symptomatic treatment used by patients during an acute attack including the reported need for medical care where invasiveness was reported from 1 to 5

**Fig. 1:**
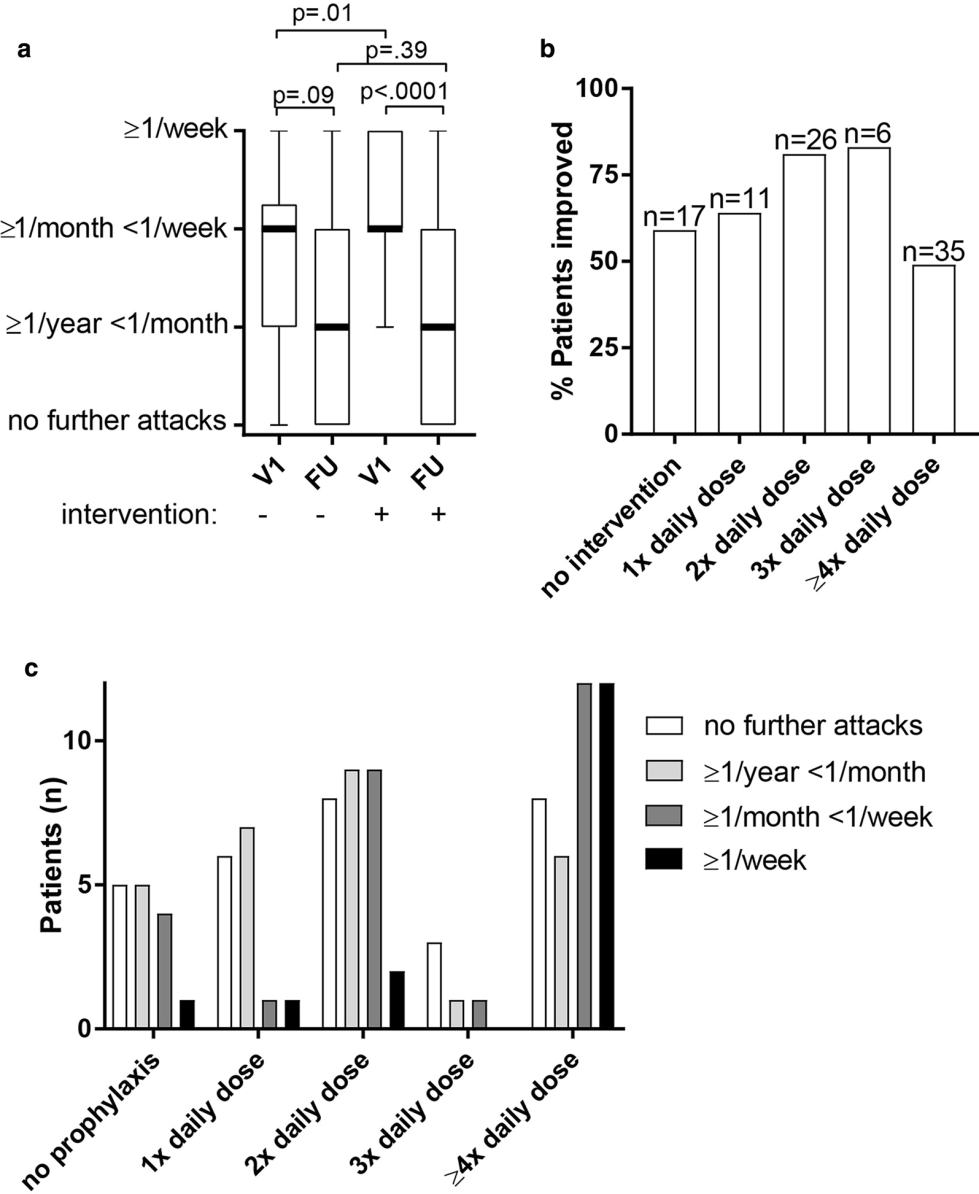
Attack frequency in relation to antihistamine prophylaxis. Attack frequencies were evaluated at maximum antihistamine dose prescribed (prior to add-on therapy) **a** box-and-whiskers-plot of attack frequency at first visit (V1) and follow-up (FU) with (+),or without intervention (−), bold line = median, Mann–Whitney and Wilcoxon test were used comparing groups respectively paired samples. **b** Percentage of patients reporting improvement per maximum dose antihistamines prescribed, improvement was defined as shift into a lower attack frequency group, n = total patients per group, patients with missing data on attack frequency could not be evaluated for improvement (n = 25). **c** Attack frequency per maximum dose prescribed

We evaluated change in attack frequency during follow-up. In 95 of the 120 patients attack frequency at first visit and during maximal prescribed antihistamine therapy was reported (missing data at first visit n = 15, missing data during follow-up n = 17, Table 2). During follow-up, attack frequency decreased significantly (*p* < 0.0001, Fig. 1a) in patients receiving intervention. A non-significant reduction in attack frequency was observed in patients not receiving prophylactic antihistamine therapy or a dose escalation (*p* = 0.09). Of these 17 patients that did not receive intervention and had data on attack frequency reported, 59% improved (Fig. 1b). This demonstrates that perceived effectiveness of antihistamine therapy may in part reflect natural disease remission. However, it should be noted that these patients in general had a lower attack frequency at first visit (*p* = 0.01, Fig. 1a) and often reported an emergency care visit or hospitalization in their history (55%) therefore, comparison with the intervention group should be made with caution.

Of the 78 patients that received prophylactic antihistamine therapy, 50 patients (64%) improved, in 28 patients (36%) attack frequency did not improve and even got worse in 4 patients (5%). It should be noted that 9 patients who did not improved only received a one- or twofold daily dose, and may have benefitted from higher doses. However, a significant proportion of patients experienced frequent attacks even at high doses of antihistamines (Fig. 1c). Among all patients receiving antihistamine prophylaxis (n = 99, including those with missing data on attack frequency at first evaluation) 40% suffered from at least 1 attack per month (Table 2), stressing that antihistamine therapy often fails to fully suppress symptoms.

Fifty (42%) patients reported that prior to the first visit they had sought immediate medical attention for at least one attack (Table 2). 7 patients reported admission to the hospital or intensive care because of a severe attack and one patient required intubation. During follow-up only 5 (4%) patients sought immediate medical care; 2 did not receive antihistamine prophylaxis and 3 were on ≥ fourfold daily dose. No patients were admitted to the hospital. This decreased incidence of need for acute treatment suggests relatively well controlled disease in patients on antihistamine prophylaxis.

Eleven antihistamine-refractory patients received add-on therapy and had their attack frequency reported. Nine patients received omalizumab: 7 of 9 patients improved. 5 went into full remission, 1 improved to < 1 attack per month. In contrast, 3 patients had ≥ 1 attack per month despite omalizumab therapy, indicating moderate responsiveness in one patient that went from weekly to monthly attacks and no response in the other 2 cases.

## Discussion

Overall, the majority of our cohort (64%) improved under prophylactic therapy with antihistamines. However, 40 (40%) patients still experienced ≥ 1 attack per month despite treatment. Previous studies reported that 15–16% of idiopathic angioedema patients were ‘non-histaminergic’ i.e. were antihistamine-refractory, compared to 36% within the current study. Notably, in these two previous studies, lower doses of antihistamines were prescribed (up to twofold daily dose) [6, 7]. It was previously observed that chronic spontaneous urticaria patients may benefit from a fourfold daily dose or even higher [8]. Differences in prevalence of antihistamine-refractory patients may be explained by different definitions of improvement. Clinical evaluation of angioedema is complicated by variability in natural course of disease. In addition, antihistamine treatment was often (46%) already started in primary or secondary care. Moreover, patients that only came for an initial visit but were then loss to follow-up, possibly due to good response, were excluded. This could potentially contribute to an over-representation of antihistamine-refractory patients in our cohort.

Our findings underline the urgent need for additional treatment options in idiopathic angioedema as monotherapy with antihistamines is often insufficient. As the underlying mechanism of idiopathic angioedema is unknown add-on therapy is based on trial-and-error. In this study, add-on therapy with omalizumab, a monoclonal antibody targeting IgE, resulted in complete disease remission in 5 out of 9 patients. So far, case reports describe a total of 23 patients with antihistamine refractory idiopathic angioedema on omalizumab that all showed complete remission [9]. Alternatively, other case reports describe successful use of therapy commonly prescribed in patients with hereditary angioedema; such as C1-esterase inhibitor concentrate, bradykinin receptor antagonist and kallikrein inhibition [10]. Hence, these therapies constitute potential treatment options for antihistamine-refractory patients but their efficacy should be further investigated in clinical trials.

In conclusion, in this retrospective study we observed a high incidence of anti-histamine refractory patients. This stresses further efforts in exploring novel treatment options for idiopathic angioedema.

## Data Availability

Contact the corresponding author H Röckmann for an anonymized database of this study.

## Abbreviation

ACE: angiotensin converting enzyme
